# The Doctor and His Work

**Published:** 1913-03

**Authors:** 


### Reviews of Books.

**The Doctor and his Work.** By CHARLES J. WHITBY, M.D. Cantab. Pp. 235. London: Stephen Swift & Co. Ltd. 1912. Price, 3s. 6d. net.

Dr. Whitby gives us, in this readable little book, what may be styled his thoughts on the medical profession.

He discusses the positions of physicians, surgeons, specialists, lady doctors, pharmacists, etc., in the ranks of medicine, and inclines to honour the general practitioner who makes a speciality of knowing the constitutions of his patients, as the most important member.

He is very severe (and we sympathise with him) upon modern quack advertisements, and a paragraph on this subject may be quoted as indicating his opinions, and as a sample of his somewhat trenchant style. He says (p. 125): "Revolting pictures of blotched and pimply faces; pseudo-scientific dissertations; anatomical obscenities; portraits of blatant American auctioneers masquerading as professors of this, that, or the other; frock-coated gymnasts lecturing to a roomful of open-mouthed doctors—these are but a few choice specimens of the blasphemous banalities which disfigure the pages of every journal of the hour," etc.

The chapter on "Medicine as a Guild" is thoughtfully written, and contains some judicious remarks on the retention of voluntary hospitals.

Dr. Whitby recognises the changes that are inevitable in medical practice and teaching, especially the new pathology introduced by Ehrlich, Wright and others.

Eugenics, homœopathy, spiritualism and other dangerous regions are fearlessly entered, and if the author cannot guide us through these, he has at least given us his opinion upon them.

The author, in fact, is not content even with these territories, but takes us into the unknown lands of speculative philosophy. Here, however, the timid reviewer cannot follow him, and must leave his interesting chapter on "The Doctor as Priest and Philosopher" to the readers (and he hopes they will be many) of this book.

